# Editorial: AI and Healthcare Financial Management (HFM) towards sustainable development

**DOI:** 10.3389/frai.2022.1096496

**Published:** 2022-12-02

**Authors:** Ananth Rao, M. V. Manoj Kumar, Nanda Kumar B. V. S. Sashtry, Immanuel Azaad Moonesar, Arkalgud Ramaprasad, Alicia Núñez, B. Annappa, Karan Bhanot, Wathiq Mansoor

**Affiliations:** ^1^Dubai Business School, University of Dubai, Dubai, United Arab Emirates; ^2^Dubai Business School, Mohammed Bin Rashid School of Government, Dubai, United Arab Emirates; ^3^Department of Information Science Engineering, Nitte Meenakshi Institute of Technology, Bangalore, Karnataka, India; ^4^Department of Public Health, M. S. Ramaiah Medical College, Bangalore, Karnataka, India; ^5^Department of Informatics, University of Illinois at Chicago, Chicago, IL, United States; ^6^Department of Informatics, University of Chile, Santiago, Chile; ^7^Department of Computer and Informations Science, National Institute of Technology Karnataka, Mangalore, Karnataka, India; ^8^Department of Finance, University of Texas at San Antonio, San Antonio, TX, United States; ^9^Department of Engineering and Information Systems, University of Dubai, Dubai, United Arab Emirates

**Keywords:** healthcare investments, Sustainable Development Goal 3, telemedicine, data management algorithm, machine learning

The World Health Organization's (WHO) Global Health Security (GHS) index of 40.2/100 indicates that almost every country has critical healthcare gaps in prevention, detection, reporting, rapid response, the capacity of the healthcare system, compliance with international standards, risk situations, and health financing for sustainable development. The amount of money people spend on healthcare out-of-pocket depends on how serious their disease is and how much money they make as a family. Healthcare expenses paid out-of-pocket are closely correlated with household income (including transfers), savings, or loans. If the household pays, such expenditure results in healthcare inequities.

With this background, the Research Topic, “*AI and healthcare financial management towards sustainable development*,” has been put forward in Frontiers in Artificial Intelligence to develop solutions to minimize the gaps by soliciting detailed manuscripts from the multi-disciplinary research community. Over the last 9 months, 15 manuscripts were submitted by diverse scholars and 10 articles were accepted for publication after rigorous peer review. Of the 10, four were research-based, four were conceptual, and two were review articles. This editorial note summarizes how each article helped to achieve the scope of the special topic and in turn, the United Nation's (UN) Sustainable Development Goal 3 (SDG-3).

A hybrid-based knowledge model for recording, storing, indexing, and querying African traditional herbal medicine, ATHMed, is investigated in the study (Devine et al.). The authors extract ATHMed data using ML and ontology. The framework employed a multi-word search pattern and a corpus that has been semantically tagged. The authors gather initial data and develop an ML technique for processing, storage, and retrieval to minimize SDG-3 gaps in the African community.

In the study (Manoj Kumar, Sastry et al.), two sub-indicators of SDG 3.8, access to high-quality (SDG 3.8.1) and affordable healthcare (SDG 3.8.2) are examined for ensuring universal health care (UHC) in three economic blocks: the developing Gulf Cooperation Council (GCC), developing countries Brazil, Russia, India, China, and South Africa (BRICS), and the developed countries of Australia, the UK, and the USA. The authors use the WHO Global Health Indicator database and UHC periodical surveys and find that the ML Random Forest Tree method is superior to the OLS model in terms of lower RMSE. ML Random Forest Tree predicts that private health expenditure, out-of-pocket spending per capita in US dollars, and voluntary health insurance as a percentage of current health expenditure, impact UHC. The study has ramifications for financial and health policies including low-cost social health insurance for the underprivileged in the developing economic blocks.

The study (Rao, Manoj Kumar et al.) uses weekly time-series data from January 2003 to December 2020 to predict first-time investor sentiments (IS) to attract investments for the growth of the health sectors of India, mainland China, and the UAE. An ANN design better mimicked investor cognitive behavior than the logistic model. Current health spending as a proportion of GDP, the USA IS predictor—spread, and GDP—annual growth percent factors influenced emerging nations' IS behavior. The study findings imply that these emerging nations' healthcare sectors have significant investment opportunities for achieving the 2030 SDG-3 and SDG-8 targets.

The scope of the financial market, which produces around 1.145 trillion megabytes (MB) of data per day (including health data), is examined in the article (Abdul Razak et al.). Massive data system analysis improved family living standards, stabilized societal activities, and enhanced environmental criteria for sustainable development. The article uses the sliding window approach and random forest algorithm to stabilize the behavior. The proposed approach provides promising results in terms of accuracy in detecting concept drift over the state of existing drift detection methods like one-class drift detection (OCDD), adaptive windowing (ADWIN), and the Page-Hinckley test.

The article (Atalla et al.) suggests autonomous intelligent healthcare prevention tools to assist multimorbid elderly patients in monitoring, anticipating, and responding to health status by alerting doctors and patients to lower unexpected health complications in real-time.

The UN's 2030 SDGs, India's National Health Policy, and the UAE's Ministry of Health Policy challenges, all call for a digital health ecosystem as outlined in the article (Manoj Kumar, Patil et al.). SDG Goal 1 and its connected purposes are the basis for virtual consultations, telemedicine, virtual storage, and virtual communities. SDGs 2 and 3 monitor and analyze PHC and POC data. In rural, urban, and remote populations of the UAE and India, the concept augments the PHC system with ICT-based interventions, to improve patient health outcomes.

The article (Manoj Kumar, Atalla et al.)_finds that deep learning approaches are a good, practical, and economical diagnostic tool for COVID-19. This research shows the least expensive and most reliable imaging strategy for predicting infections, by comparing COVID-19 detection methods, which have implications for reducing health insurance costs.

The article (von Ulmenstein et al.) conceptualizes a novel AI model to access medical information that threatens to exacerbate adverse selection in the health insurance market, by conducting an interdisciplinary conceptual analysis to examine how this risk could be mitigated, taking into account legal, ethical, and economic considerations. The authors propose that these health hazards cannot be disregarded in future medical applications of AI forecasting and must be handled structurally.

The study (Abdul Razak and Nirmala) conceptualizes many forms of concept drift problems in healthcare data streams and summarizes the available statistics and ML methodologies for addressing concept drift. The authors also emphasize the use of deep learning algorithms for concept drift identification and provide a summary of the various healthcare datasets utilized for concept drift detection in the categorization of data streams.

The authors of the article (Rao, Sastry et al.) study the role of AI-based cost optimization in India's universal colorectal carcinoma (CRC) prevention campaigns. AI-based detection tools and CADx systems are the way forward in CRC prevention. They reduce CRC and ADR and India's cost-effectiveness is shown to have improved. AI may change CRC screening by determining the colonoscopy monitoring interval based on morphological and clinical factors. This might help reallocate Resources and avoid repeat treatments for low-risk patients. This strategy might save money without compromising pharmaceutical safety or effectiveness. AI-based polyp detection and characterization may enhance CRC prevention.

In conclusion, the authors have done a pretty good job of addressing the objectives of the Research Topic. A sincere thank to each one of them for their contribution.

## Author contributions

All authors listed have made a substantial, direct, and intellectual contribution to the work and approved it for publication.

## Conflict of interest

The authors declare that the research was conducted in the absence of any commercial or financial relationships that could be construed as a potential conflict of interest.

## Publisher's note

All claims expressed in this article are solely those of the authors and do not necessarily represent those of their affiliated organizations, or those of the publisher, the editors and the reviewers. Any product that may be evaluated in this article, or claim that may be made by its manufacturer, is not guaranteed or endorsed by the publisher.

